# Revacept, an Inhibitor of Platelet Adhesion in Symptomatic Carotid Artery Stenosis: Design and Rationale of a Randomized Phase II Clinical Trial

**DOI:** 10.1055/s-0040-1721078

**Published:** 2020-11-30

**Authors:** Klaus Gröschel, Timo Uphaus, Ian Loftus, Holger Poppert, Hans Christoph Diener, Jenny Zobel, Götz Münch

**Affiliations:** 1 Department of Neurology, University Medical Center of the Johannes Gutenberg University Mainz, Mainz, Germany; 2 Department of Vascular Surgery, St George's University Hospitals NHS Foundation Trust, London, United Kingdom; 3 Department of Neurology, Klinikum rechts der Isar, Technical University Munich, Munich, Germany; 4 Institute for Medical Informatics, Biometry and Epidemiology, Medical Faculty of the University Duisburg-Essen, Essen, Germany; 5 advanceCOR, Munich, Germany

**Keywords:** Revacept, platelet inhibitor, transient ischemic attack, carotid stenosis, stroke

## Abstract

Patients with stroke or transient ischemic attacks (TIAs) and internal carotid artery stenosis harbor an increased risk of recurrent stroke especially within 2 weeks after the first event. In addition, the revascularization procedure itself (carotid endarterectomy [CEA] or carotid artery stenting [CAS]) is associated with both clinically apparent and silent brain infarctions, mainly caused by the embolic nature of the ruptured carotid plaque. The glycoprotein VI (GPVI) fusion protein Revacept is a highly specific antithrombotic drug without direct inhibition of systemic platelet function that might reduce periprocedural distal embolization from the vulnerable ruptured plaque located at the internal carotid artery. By shielding collagen at the site of vascular injury, Revacept inhibits plaque-mediated platelet adhesion and aggregation, while not directly affecting systemic hemostasis. In this phase II study, 158 patients with symptomatic carotid artery stenosis with recent TIA or stroke were randomized to receive a single dose of either Revacept (40 or 120 mg) or placebo. All patients were on standard secondary preventive therapy (statins and platelet inhibition) and underwent CEA, CAS, or best medical therapy according to current guidelines. The efficacy of Revacept was evaluated by exploratory assessment of new diffusion-weighted imaging lesions on magnetic resonance imaging after the revascularization procedure; a combination of cardiovascular events (ischemic and hemorrhagic stroke, TIA, myocardial infarction, or coronary intervention) and bleeding complications served to assess clinically critical patients' outcome and safety. This exploratory phase II randomized, double-blind clinical trial provides valuable insights on the safety, tolerability, and efficacy of Revacept in patients with symptomatic carotid artery stenosis.

## Introduction

Patients suffering from transient ischemic attack (TIA) or stroke caused by carotid artery stenosis are at a 21% increased risk of experiencing recurrent strokes within the 14 days after the initial event. 1 2 Therefore, current guidelines advise a timely revascularization procedure (carotid endarterectomy [CEA] or carotid angioplasty and stenting [CAS]) to reduce the risk of recurrent brain infarctions. 3 The increased risk for subsequent ischemic events is caused by the underlying atherosclerosis, generating arterioarterial emboli from the ruptured plaque. Moreover, the revascularization procedure itself can cause emboli at the site of carotid artery stenosis and at the iatrogenic thrombogenic surface (stent or surgical neointima). In a meta-analysis of three carotid stenting clinical trials (namely EVA-3S, SPACE, and ICSS), 4 the risk of periprocedural ischemic stroke was 5.8% after CEA and 8.9% after CAS. Although current antiplatelet agents are able to reduce the risks for recurrent stroke during and after CEA 5 and CAS, 6 their use is associated with major and potentially life-threatening bleeding complications. 7

Patients with acute stroke and TIA due to carotid artery stenosis are not only at high risk of recurrent ischemic events but also at high risk of bleeding complications including intracranial hemorrhage. 5 Therefore, antiplatelet monotherapy is currently the guideline conform treatment for these patients. 3 Several trials with intensified antiplatelet therapy in patients with TIA and stroke due to carotid stenosis failed because the improvement in anti-ischemic protection was counterbalanced by increased bleeding rates, which severely affected the clinical outcome in these patients. 5

There is therefore a high medical need for efficient inhibition of thrombus formation at the vulnerable plaque without affecting general hemostasis, thereby potentially avoiding increased bleeding rates, especially in patients with previous stroke or TIA. Revacept is a protein comprised of an Fc (fragment crystallizable) fragment fused to the endogenous platelet collagen receptor glycoprotein VI (GPVI). Collagen is the most efficient facilitator of plaque-mediated thrombosis 8 while GPVI is the most abundant collagen receptor on platelets. 9 Revacept binds to its ligand collagen (and to other extracellular matrix proteins including fibronectin, vitronectin, and laminin on atherosclerotic plaques 10 11 ), thereby preventing circulating thrombocytes from binding to collagen exposed by the injured plaque. Revacept inhibits binding of von Willebrand factor to collagen, thus impacting local platelet activation via glycoprotein Ib. 12 As Revacept does not directly bind to platelets or block platelet surface receptors, it does not impair general thrombocyte activity in animal models 13 or in healthy humans in a phase I study. 14 As a unique feature, and in contrast to currently available antiplatelet drugs, Revacept does not directly interfere with the overall physiological activity of platelets. 15 Therefore, Revacept provides a promising therapeutic strategy with lesion-directed inhibition of thromboembolization at the site of the acute ruptured plaque that does not compromise systemic hemostasis. By masking collagen exposure to the blood stream at the site of atherosclerotic plaques rather than directly inhibiting thrombocytes, local thrombosis can be prevented without jeopardizing systemic platelet functions or coagulation. This novel strategy distinguishing between pathological thrombosis and physiological hemostasis was investigated in the Revacept/CS/02 study.

As the first patient study with Revacept, the study focused on patients with recent TIA or stroke undergoing CEA or CAS for removal of the carotid artery stenosis. Revacept seemed highly suitable to address the plaque- and intervention-mediated thrombosis based on the mechanism of action and the available preclinical animal data.

### Safety and Efficacy of Revacept in Animal and Ex Vivo Models

The mode of action of Revacept was studied in animal models in great detail. 11 16 17 18 When arterial lesions were induced to the carotid artery in mice models of atherosclerosis, Revacept was effective at preventing platelet adhesion and thrombus formation at these sites without affecting bleeding time. Furthermore, bleeding times were also not increased when Revacept was combined with conventional antiplatelet agents such as aspirin, clopidogrel, or heparin. 18 Further preclinical investigation showed that Revacept strongly inhibits human plaque-induced thrombosis in ex vivo superfusion models using human patient blood and plaques gained during carotid surgery. 19 Moreover, Revacept is characterized by a promising pharmacovigilance profile with no signs of toxicities or aberrant immune activation detected in preclinical animal studies even after repeated dosing.

## Study Objectives, Design, and End Points

This clinical study evaluated the safety and efficacy of Revacept in patients at high risk of arterial thrombosis with unstable or ruptured atherosclerotic plaques at the site of the carotid artery. To this end, the target group were patients with symptomatic carotid artery stenosis undergoing surgical, endovascular interventions, or best medical therapy (BMT) as a guideline-conform treatment to reduce future ischemic events. 3 It was hypothesized that Revacept can reduce the generation of naturally occurring arterial thrombosis by the underlying cerebrovascular disease as well as due to periprocedural iatrogenic ischemic events caused by platelet thrombi arising from the thrombogenic surface of the ruptured plaque or either the stent or the neointima generated by the surgical procedure during CEA and CAS. Therefore, Revacept was tested as an early secondary prophylaxis medication to diminish arterial thrombosis and consecutive ischemic events with the overall aim of specific plaque-selective platelet inhibition without additional bleeding complications due to a general platelet dysfunction.

**Fig. 1:**
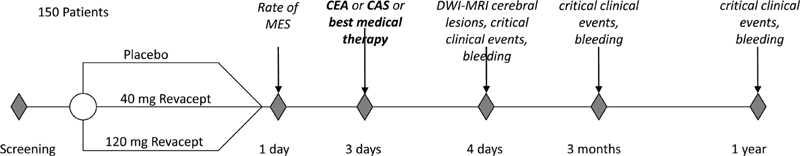
Timeline for the study protocol of patients with symptomatic carotid artery disease. Brief overview of the timeline of the study protocol of the Revacept/CS/02 study investigating the effects of Revacept in patients with symptomatic carotid artery stenosis. Study visits are indicated by diamonds; study procedures or evaluation of critical end points are listed. CAS, carotid angioplasty and stenting; CEA, carotid endarterectomy; MES, microembolic signals; MRI, magnetic resonance imaging.

Patients suffering from TIA, amaurosis fugax, or ischemic stroke received a single dose of trial medication, underwent either CEA, CAS, or BMT and were followed up clinically 1 day, 3 days, and 3 months after treatment, and by a telephonic interview at 12 months ( Fig. 1 ).

Efficacy end points included whether the incidence or rate per hour of preoperative microembolic signals (MES) as detected by transcranial Doppler examination is reduced compared with prior to drug administration, assessment of the neurological status (with National Institute of Heath Stroke Scale, NIHSS), and cerebral lesion analysis by diffusion-weighted imaging with magnetic resonance imaging (DWI-MRI) for the assessment of symptomatic and asymptomatic brain infarctions. Moreover, clinical end points were evaluated before treatment and 1 day, 3 days, 3 months, and 12 months after treatment. These end points included the rate of all causes of death, the rate of stroke-related death, and the occurrence of TIA, amaurosis fugax, or stroke including hemorrhagic stroke. In addition, cardiovascular outcome including myocardial infarction or re-intervention within 3 and 12 months was assessed.

Safety end points were summarized by treatment group and included vital signs, electrocardiogram (ECG) parameters, antidrug antibody titers, and adverse events (AEs) including wound healing complications, laboratory abnormalities, and the use of concomitant medication. Hemostasis was also closely monitored by assessing laboratory parameters indicating thrombocytopenia, bleeding events according to the Randomized Evaluation of Long-Term Anticoagulant Therapy (RE-LY) study group criteria, 20 in vitro platelet function with collagen, thrombin receptor activating peptide (TRAP)-, and adenosine diphosphate (ADP)-mediated platelet aggregation, and in vitro bleeding time by platelet function assay (PFA)-100/PFA-200 where technically feasible. AEs were continuously recorded and overseen by an independent Data Safety Monitoring Board. Details of the efficacy and safety end points are summarized in Table 1 .

**Table 1:** Study objectives and end points of the exploratory Revacept/CS/02 study in patients with symptomatic carotid artery stenosis

Efficacy end points	Safety objectives
• To evaluate whether the incidence of preoperative microembolic signals (MES) is reduced in patients with symptomatic carotid artery stenosis who have been treated with Revacept plus antiplatelet monotherapy (aspirin or clopidogrel) vs. antiplatelet monotherapy alone (placebo). MES will be assessed by transcranial Doppler (TCD) examination (before and after treatment). • Rate of MES per hour (before and after treatment). • Assessment of neurological status (NIH stroke scale). • Cerebral lesion analysis by DWI-MRI and correlation to neurological status (before and after treatment). • Clinical end points will be summarized cumulatively, i.e., before treatment, 1 and 3 d after treatment, at 3 and 12 mo. The following end points will be recorded: ○ Rate of all causes of death. ○ Rate of stroke-related death. ○ Any TIA, amaurosis fugax, or stroke including hemorrhagic stroke. • Assessment of cardiovascular outcome including myocardial infarction and re-intervention up to 3 and 12 mo.	• Safety objectives will be summarized by treatment group and include: • Vital signs. • ECG parameters. • Antidrug antibody titers. • Reporting AEs including wound healing complications, laboratory abnormalities, and use of concomitant medication. • Hemostasis will be closely monitored by assessing: ○ laboratory parameters indicating thrombocytopenia. ○ where feasible: in vitro platelet function with collagen, TRAP, and ADP-mediated platelet aggregation and in vitro bleeding time by PFA-100/PFA-200. • Bleeding complications (major) according to the RE-Ly study group criteria or reported as AE by clinical investigator (minor).

Abbreviations: ADP, adenosine diphosphate; AE, adverse event; DWI-MRI, diffusion-weighted magnetic resonance imaging; ECG, electrocardiogram; MES, microembolic signals; PFA, platelet function assay; TCD, transcranial Doppler; TIA, transient ischemic attack; TRAP, thrombin receptor activating peptide.

An overview of the protocol-related procedures is given in Table 2 .

**Table 2:** Overview of procedures of the phase II study Revacept/CS/02 in patients with symptomatic carotid artery stenosis

		Screening	Randomization	Treatment (T)	T +24 h ( ± 22 h)	T + 3 d (−69 h/+ 5 d	CEA + 24 h ( ± 24 h)	Follow-up	
								T+ 3 m ( ± 1 m)	T + 12 m ( ± 1 m)
Procedure	Visit	1	–	2	3	4	5	6	7
Informed consent		X							
Randomization			X						
Study medication (Revacept or placebo)				X					
CEA/CAS						X			
CEA/CAS outcome							X		
Anamnesis		X							
Concomitant medication		X		X	X	X	X	X	
Physical examination		X		X	X	X	X	X	
Adverse events				X	X	X	X	X	
Modified Rankin Scale, Barthel index				X				X	
NIH stroke scale				X			X	X	
Clinical outcome								X	X
TCD		X			X				
Electrocardiogram		X			X		X	X	
DWI-MRI		X					X		
Laboratory tests	Biochemistry	X			X		X	X	
	Hematology/Bleeding	X			X		X	X	
	Coagulation	X			X		X	X	
	Urinalysis	X			X		X	X	
	In vitro bleeding time (PFA100/PFA200) and aggregation			X	X	X		X	
	Pregnancy test	X							
	Pharmacokinetics (selected patients)			X	X	X		X	
	Antidrug antibodies			X				X	

Abbreviations: CAS, carotid angioplasty and stenting; CEA, carotid endarterectomy; DWI-MRI, diffusion-weighted magnetic resonance imaging; PFA, platelet function assay; TCD, transcranial Doppler.

## Patient Population

The trial was started in Germany in 2012 and extended to the United Kingdom in 2013. A total of 160 patients were recruited from 16 study centers. Patients were included if they had signed written informed consent, were at least 18 years old, and were diagnosed with symptomatic (TIA, amaurosis fugax, or ischemic stroke within the last 30 days) extracranial carotid artery stenosis (lesions with ≥50% stenosis according to European Carotid Surgery Trial [ECST] criteria). Major exclusion criteria were an NIHSS score >18, intracerebral hemorrhage, cardiac cause of embolization as well as thrombocytopenia, bleeding diathesis or coagulopathy. Oral anticoagulation or dual antiplatelet therapy with aspirin or clopidogrel and other purinergic receptor Y _12_ (P2Y) inhibitors at screening were also prohibited.

## Randomization and Treatment

Patients were assigned to a treatment group in a double-blind manner using the web-based randomization system “Randomizer” provided by the Institute for Medical Informatics, Statistics and Documentation of the Medical University of Graz. The system used a minimized randomization method to balance potential prognostic factors between individual treatment arms. Stratification factors included antiplatelet therapy (aspirin or clopidogrel) prior to screening, statin therapy prior to screening, and the degree of stenosis (50–70% vs. >70%; ECST criteria). The study drug (40 mg Revacept, 120 mg Revacept, or placebo) was administered once per patient by intravenous (IV) infusion for 20 minutes using an in-line filter and syringe pump. All patients remained under the guideline conform secondary preventive therapy and underwent CEA, CAS, or BMT without any delay due to the study protocol. Changes in concomitant medication were at the discretion of the individual Investigator as required by the clinical situation of the patient.

## Sample Size Calculation

Sample size estimation was conducted based on the Clopidogrel and Aspirin for Reduction of Emboli in Symptomatic Carotid Stenosis (CARESS) study, which assessed the incidence of MES as the primary end point when comparing mono versus dual antiplatelet therapy in patients with recently symptomatic carotid artery stenosis. 21 Based on these results, it was estimated that the treatment efficiency of Revacept could be demonstrated with a power of 80% when 50 patients were allocated to each of the three treatment arms using a two-sided Fisher's exact test at a significance level of *α* = 0.05. As the sample size calculation was based on MES reduction, only patients presenting with MES as detected by vascular ultrasound were originally eligible to participate in the clinical trial.

## Rationale for Dose Finding

Two doses of Revacept were investigated in patients with carotid artery stenosis and recent TIA or ischemic stroke. The doses of 40 and 120 mg for the effective inhibition of plaque-mediated thrombosis were derived from the phase I study in healthy volunteers. 14 40 mg was the first dose to effectively inhibit collagen-mediated platelet aggregation after IV infusion in healthy men. 120 mg Revacept was chosen as the higher dose to maintain some safety margin below the maximally tested dose in the previous phase I study. A safety margin was introduced because drug interactions with concomitant antiplatelet agents could potentiate the bleeding complications of Revacept. Moreover, comorbidities, particularly recent strokes, make patients more vulnerable to bleeding complications especially intracranial bleeding. 16 Thus, in this first patient study with Revacept, 40 and 120 mg were elected as safe doses to show efficacy while also taking safety into account.

## Change in the Conduct of Study during Recruitment

During the course of the study, fewer patients than anticipated presented with MES upon screening which was the overt reason for screening failures. An initial screen failure rate of 78% thus greatly delayed recruitment. Following the recommendation of the Data Safety Monitoring Board, the prerequisite for MES at screening was therefore discontinued. In the original study protocol, the reduction in MES was the primary end point for efficacy. After removing positive MES as an inclusion criterion, the reduction in MES could no longer be investigated. Consequently, the strategy of the study was changed to an exploratory trial design while maintaining the existing and predefined end points. New DWI lesions on MRI, any strokes (cerebral ischemia, cerebral hemorrhage, TIA), other ischemic events (myocardial infarction or coronary intervention), and bleeding complications as originally defined in the study protocol were therefore investigated in an exploratory manner. The necessary amendment was approved by the appropriate national regulatory authorities (in Germany: Bundesinstitut für Arzneimittel und Medizinprodukte [BfArM] and the United Kingdom: Medicines and Healthcare products Regulatory Agency [MHRA]) and received positive opinions by both national corresponding ethics committees (Ethikkommission TU Munich and National Research Ethics Service [NRES] Cambridge South).

## Statistical Analysis Plan

All data handling and analyses were performed by an independent data analysis team (DSH statistical services, Rohrbach, Germany). All clinical events were recorded on paper-based case report forms (CRF). All data on CRF was then compiled in an SAS data bank (SAS Institute, Cary, North Carolina, United States). Unblinding and subsequent analysis of safety and efficacy was possible only after data lock of the SAS data bank. Baseline data were presented in a descriptive way for safety, intention-to-treat, and per-protocol analysis sets. All statistical tests were performed two-sided and were interpreted in a descriptive, exploratory way. Tests for numerical data assumed normality and were performed using analysis of variance (ANOVA). Furthermore, normality of distribution was tested based on goodness of fit tests. If no normality was assumed, sensitivity analyses were performed using the Wilcoxon test. Two-sided confidence intervals were displayed for important variables.

## Efficacy

Comparison of the neurological status (NIHSS) between groups was performed using the Mantel-Haenszel Chi-square test; the total NIHSS score was compared between groups using ANOVA.

The change in number as well as percent change in DWI lesions before treatment versus after CEA/CAS was compared using ANOVA. Fisher's exact test was used to compare incidence of patients with DWI lesions, incidence of patients with new DWI lesions, and rate of patients with reduced DWI lesions. The number of new DWI lesions was analyzed accordingly. Correlation of cerebral lesions to functional neurological status based on Modified Rankin Scale was assessed using Pearson correlation coefficient. Subgroup analysis focusing on patients with more severe carotid artery stenosis (>70% ECST) and those not receiving concomitant antiplatelet medication (aspirin or clopidogrel) at admission to the hospital was performed.

The originally planned reduction in the incidence of preoperative MES in patients could not be analyzed reliably after the change in the study protocol and will therefore be reported in a descriptive manner.

The cumulative clinical end point rate of all causes of death, any TIA, or stroke including hemorrhagic stroke, rate of myocardial infarctions, and coronary re-intervention was summarized by means of frequency tables; treatment groups were compared using Fisher's exact test. Combined clinical end points for ischemic events (myocardial infarction and ischemic stroke/TIA) and the rate of rescue medication (additional antiplatelet co-medication) during the study were analyzed using the Cochran-Mantel-Haenszel procedure. Subgroup analysis focusing on patients with more severe carotid artery stenosis (>70% ECST) and those not receiving concomitant antiplatelet medication (aspirin or clopidogrel) at admission to the hospital was also performed.

## Safety

Results for vital signs and changes from baseline were summarized by means of descriptive statistics and compared between treatment groups using the Wilcoxon test. Abnormal results of physical examinations not present at baseline and ECG parameters were summarized by means of frequency tables.

As the main expected AEs of intensified antiplatelet medication with Revacept, special focus for safety considerations was on bleeding complications. Revacept (or placebo) was always added to the baseline of antithrombotic medication. All bleeding events were recorded as AEs at discretion of the local investigator. Major bleeding was defined according to the RE-LY definitions 20 as a reduction in the hemoglobin level of at least 20 g per liter, transfusion of at least two units of blood, or symptomatic bleeding in a critical area or organ including intracranial hemorrhage.

AEs were categorized by primary system organ class and Medical Dictionary for Regulatory Activities (MedDRA) preferred term as coded using the MedDRA dictionary. An overview table was presented with the number (and percentage) of patients with at least one AE, serious adverse events, AEs leading to treatment discontinuation, and drug-related AEs. The percentage of patients with at least one AE was presented in frequency tables by reported AE. Laboratory values were evaluated in an exploratory manner using descriptive methods of statistics. Generated *p* -values were interpreted in an exploratory way.

### Ethical Considerations

This study was performed in accordance with the Declaration of Helsinki (1996), Good Clinical Practice as defined by the International Conference on Harmonisation, and in agreement with the ethical principles underlying the European Directive 2001/20/EC and applicable local laws and regulations, in particular, the German GCP-Verordnung and Arzneimittelgesetz as well as The Medicines for Human Use (Clinical Trials) Regulations 2004 (United Kingdom).

A Data Safety Monitoring Board was established with neurologists and cardiologists experienced in the conduct of clinical studies together with a bio-statistician. Since in this phase II study Revacept was applied for the first time to patients, special cautions were taken. The first 10 patients were dosed sequentially; a data safety analysis was performed after each patient and a data review was conducted after completion of the first 10 patients prior to starting parallel recruitment.

## Conclusion

We consider the clinical situation of patients with carotid artery stenosis and recent TIA or ischemic stroke best suited to test the novel plaque-specific potencies of Revacept. The underlying cause for the carotid artery stenosis to become symptomatic is most likely an unstable atherosclerotic plaque with exposure of the subendothelial structures such as collagen, subsequent thrombocyte activation, and cerebrovascular embolization. In addition, the guideline conform revascularization procedure by CAS and CEA may also cause additional exposure of the subendothelium leading to further distal embolization. In previous ex vivo and animal studies, which closely resemble the situation in patients with symptomatic carotid stenosis Revacept proved to effectively prevent local thrombus formation. Therefore, this patient study was the human translation of previous animal data.

The study protocol including the imaging of cerebral ischemia with DWI-MRI allowed the effects on thrombus inhibition with respect to the resulting tissue damage within the brain to be assessed. From this, a judgment can be made regarding efficacy to prevent thromboembolic events by directly assessing the end-organ damage in the patient's brain. Moreover, DWI-MRI lesions are associated with recurrent stroke in the future and worse clinical outcome and therefore are a surrogate end point with prognostic relevance in this patient population. 16 22 The long-term clinical effect of a single peri-interventional application of Revacept was also followed for 90 days with extension for 365 days to assess the overall long-term clinical outcome. Important ischemic end points such as stroke and myocardial infarction provide insight on the antithrombotic potency of Revacept. Moreover, bleeding complications were taken into consideration as the most expected side effect of any antithrombotic therapy and having major impact on the further patient outcome and prognosis. 17

This international, multicenter, randomized, double-blind, and placebo-controlled study with parallel groups generates valuable data on the safety and efficacy of Revacept in symptomatic carotid artery stenosis, and thereby providing an important basis for further development of this potent and unique therapeutic strategy for the management of a plethora of diseases caused by atherothrombosis.

## References

[1] Lovett J K Coull A J Rothwell P M Early risk of recurrence by subtype of ischemic stroke in population-based incidence studies Neurology 2004 62 04 569 573 14981172 10.1212/01.wnl.0000110311.09970.83

[2] Fairhead J F Mehta Z Rothwell P M Population-based study of delays in carotid imaging and surgery and the risk of recurrent stroke Neurology 2005 65 03 371 375 16087900 10.1212/01.wnl.0000170368.82460.b4

[3] ESC Scientific Document Group Aboyans V Ricco J B Bartelink M EL 2017 ESC Guidelines on the Diagnosis and Treatment of Peripheral Arterial Diseases, in collaboration with the European Society for Vascular Surgery (ESVS): document covering atherosclerotic disease of extracranial carotid and vertebral, mesenteric, renal, upper and lower extremity arteries endorsed by: the European Stroke Organization (ESO)The Task Force for the Diagnosis and Treatment of Peripheral Arterial Diseases of the European Society of Cardiology (ESC) and of the European Society for Vascular Surgery (ESVS) Eur Heart J 2018 39 09 763 816 28886620 10.1093/eurheartj/ehx095

[4] Carotid Stenting Trialists' Collaboration Bonati L H Dobson J Algra A Short-term outcome after stenting versus endarterectomy for symptomatic carotid stenosis: a preplanned meta-analysis of individual patient data Lancet 2010 376(9746):1062 1073 20832852 10.1016/S0140-6736(10)61009-4

[5] Jones D W Goodney P P Conrad M F Dual antiplatelet therapy reduces stroke but increases bleeding at the time of carotid endarterectomy J Vasc Surg 2016 63 05 1262 1.27E6 26947237 10.1016/j.jvs.2015.12.020 PMC5065102

[6] Abou-Chebl A Yadav J S Reginelli J P Bajzer C Bhatt D Krieger D W Intracranial hemorrhage and hyperperfusion syndrome following carotid artery stenting: risk factors, prevention, and treatment J Am Coll Cardiol 2004 43 09 1596 1601 15120817 10.1016/j.jacc.2003.12.039

[7] van Rein N Heide-Jørgensen U Lijfering W M Dekkers O M Sørensen H T Cannegieter S C Major bleeding rates in atrial fibrillation patients on single, dual, or triple antithrombotic therapy Circulation 2019 139 06 775 786 30586754 10.1161/CIRCULATIONAHA.118.036248

[8] Reininger A J Bernlochner I Penz S M A 2-step mechanism of arterial thrombus formation induced by human atherosclerotic plaques J Am Coll Cardiol 2010 55 11 1147 1158 20223370 10.1016/j.jacc.2009.11.051

[9] Nieswandt B Watson S P Platelet-collagen interaction: is GPVI the central receptor? Blood 2003 102 02 449 461 12649139 10.1182/blood-2002-12-3882

[10] Bültmann A Li Z Wagner S Impact of glycoprotein VI and platelet adhesion on atherosclerosis—a possible role of fibronectin J Mol Cell Cardiol 2010 49 03 532 542 20430036 10.1016/j.yjmcc.2010.04.009

[11] Schönberger T Ziegler M Borst O The dimeric platelet collagen receptor GPVI-Fc reduces platelet adhesion to activated endothelium and preserves myocardial function after transient ischemia in mice Am J Physiol Cell Physiol 2012 303 07 C757 C766 22814400 10.1152/ajpcell.00060.2012

[12] Goebel S Li Z Vogelmann J The GPVI-Fc fusion protein Revacept improves cerebral infarct volume and functional outcome in stroke PLoS One 2013 8 07 e66960 23935828 10.1371/journal.pone.0066960 PMC3720811

[13] Massberg S Konrad I Bültmann A Soluble glycoprotein VI dimer inhibits platelet adhesion and aggregation to the injured vessel wall in vivo FASEB J 2004 18 02 397 399 14656994 10.1096/fj.03-0464fje

[14] Ungerer M Rosport K Bültmann A Novel antiplatelet drug Revacept (Dimeric Glycoprotein VI-Fc) specifically and efficiently inhibited collagen-induced platelet aggregation without affecting general hemostasis in humans Circulation 2011 123 17 1891 1899 21502572 10.1161/CIRCULATIONAHA.110.980623

[15] Majithia A Bhatt D L Novel antiplatelet therapies for atherothrombotic diseases Arterioscler Thromb Vasc Biol 2019 39 04 546 557 30760019 10.1161/ATVBAHA.118.310955 PMC6445601

[16] ICSS-MRI Substudy Investigators Gensicke H van der Worp H B Nederkoorn P J Ischemic brain lesions after carotid artery stenting increase future cerebrovascular risk J Am Coll Cardiol 2015 65 06 521 529 25677309 10.1016/j.jacc.2014.11.038 PMC4323145

[17] Ndrepepa G Berger P B Mehilli J Periprocedural bleeding and 1-year outcome after percutaneous coronary interventions: appropriateness of including bleeding as a component of a quadruple end point J Am Coll Cardiol 2008 51 07 690 697 18279731 10.1016/j.jacc.2007.10.040

[18] Ungerer M Li Z Baumgartner C The GPVI-Fc fusion protein Revacept reduces thrombus formation and improves vascular dysfunction in atherosclerosis without any impact on bleeding times PLoS One 2013 8 08 e71193 23951109 10.1371/journal.pone.0071193 PMC3741334

[19] Jamasbi J Megens R T Bianchini M Differential inhibition of human atherosclerotic plaque-induced platelet activation by dimeric GPVI-Fc and Anti-GPVI antibodies: functional and imaging studies J Am Coll Cardiol 2015 65 22 2404 2415 26046734 10.1016/j.jacc.2015.03.573 PMC4452546

[20] RE-LY Steering Committee and Investigators Connolly S J Ezekowitz M D Yusuf S Dabigatran versus warfarin in patients with atrial fibrillation N Engl J Med 2009 361 12 1139 1151 19717844 10.1056/NEJMoa0905561

[21] Markus H S Droste D W Kaps M Dual antiplatelet therapy with clopidogrel and aspirin in symptomatic carotid stenosis evaluated using doppler embolic signal detection: the Clopidogrel and Aspirin for Reduction of Emboli in Symptomatic Carotid Stenosis (CARESS) trial Circulation 2005 111 17 2233 2240 15851601 10.1161/01.CIR.0000163561.90680.1C

[22] ICSS-MRI study group Bonati L H Jongen L M Haller S New ischaemic brain lesions on MRI after stenting or endarterectomy for symptomatic carotid stenosis: a substudy of the International Carotid Stenting Study (ICSS) Lancet Neurol 2010 9 04 353 362 20189458 10.1016/S1474-4422(10)70057-0

